# Ostéochondrome de fémur révélé par une lésion itérative du muscle vaste latéral: à propos d'un cas

**DOI:** 10.11604/pamj.2015.20.428.6762

**Published:** 2015-04-29

**Authors:** Jemni Sonia, Frioui Samia, Elmtawa Sahbi, Osman Walid, Khachnaoui Fayçal

**Affiliations:** 1 Service Médecine Physique Réadaptation Fonctionnelle, EPS Sahloul, Sousse- Tunisie; 2 Service Orthopédie, Hopital Sahloul, Sousse, Tunisie

**Keywords:** Exostose, ostéochondrome, déchirure musculaire, exostosis, osteochondroma, muscle tear

## Abstract

Nous rapportons le cas d'un jeune homme de 32 ans présentant un ostéochondrome de fémur droit révélé par une lésion itérative du vaste externe. Le patient consulte pour des épisodes récidivants de douleur de la cuisse droite avec impotence fonctionnelle, survenant lors d'une activité sportive et imposant son arrêt. L’échographie a montré une déchirure du vaste latéral, avec un hématome témoignant d'une lésion récente et présence de fragments osseux à proximité évoquant un arrachement osseux. Un bilan radiologique standard a montré une exostose pédiculaire à la partie supérieure de la diaphyse fémorale compliquée d'une fracture. La tomodensitométrie était en faveur d'une exostose fémorale antérieure dont les limites étaient régulières et bien corticalisées. La résection chirurgicale de la tumeur et l'examen anatomo-pathologique ont permis de confirmer le diagnostic d'un ostéochondrome.

## Introduction

Les exostoses ostéogéniques, appelées aussi ostéochondromes, correspondent à des excroissances osseuses recouvertes d'une coiffe cartilagineuse [1]. Elles sont habituellement asymptomatiques. Cependant, elles peuvent se compliquer et devenir symptomatiques.

## Patient et observation

**Figure 1:**
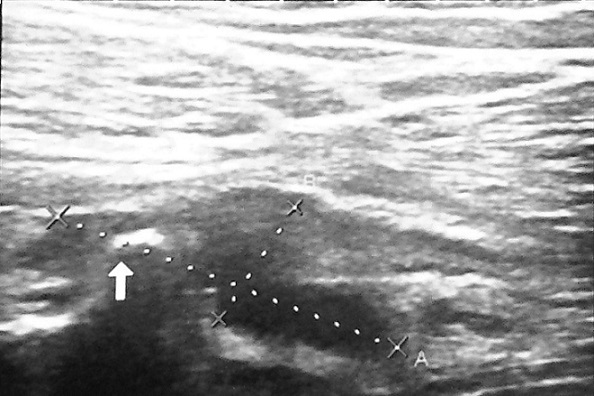
Echographie des parties molles de la cuisse droite montrant un hématome du muscle vaste latéral avec des fragments osseux au niveau de la lésion musculaire évoquant un arrachement osseux

**Figure 2:**
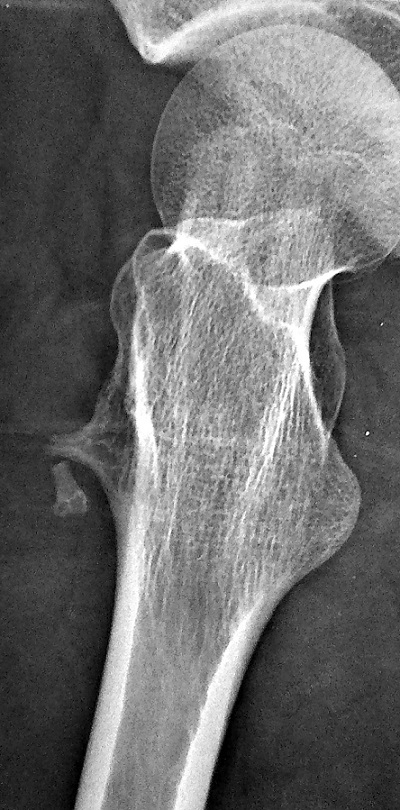
Radiographie du fémur droit montrant l'exostose pédiculée à l'extrémité supérieure de la diaphyse fémorale compliquée d'une fracture

**Figure 3:**
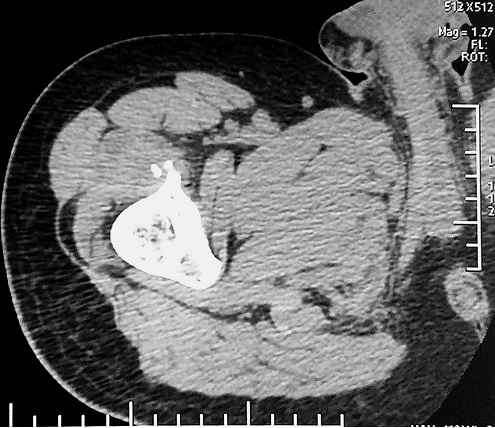
Scanner du fémur droit (coupe axiale) montrant une exostose fémorale antérieure pédiculée, en conflit avec le muscle vaste latéral et fracture

Patient âgé de 32 ans, éducateur sportif, se présente à la consultation de Médecine Physique et Réadaptation Fonctionnelle pour des douleurs de la cuisse droite, évoluant depuis une semaine, survenues lors d'un match de foot et imposant l'arrêt de l'activité physique. Le patient signale des épisodes similaires durant les deux dernières années. A l'examen physique, la marche est possible avec une légère boiterie à droite. Il n'y a pas de tuméfaction décelable à la palpation de la cuisse droite. Les amplitudes articulaires des membres inférieurs sont conservées. La contraction résistée du quadriceps droit réveille une douleur importante. Une échographie de la cuisse droite a montré une déchirure musculaire au dépend du vaste latéral avec un hématome mesurant 27 x 9 x 12 mm et présence des fragments osseux faisant suspecter un arrachement osseux (Figure 1). Un complément par une radiographie de fémur a permis d'objectiver une exostose pédiculaire à la partie supérieure de la diaphyse fémorale, compliquée d'une fracture (Figure 2). Un scanner a confirmé la présence d'exostose fémorale antérieure, dont les limites apparaissent régulières et bien corticalisées (Figure 3). Le patient a été opéré, avec une résection en bloc de la tumeur et extraction des fragments détachés. L'examen anatomopathologique a conclu un ostéochondrome. En post opératoire, le patient a bénéficié d'un programme de rééducation visant l'obtention d'une indolence, la récupération de l'extensibilité et de la force des muscles sous pelviens. A six mois post-opératoire, le patient a repris son travail sans incident.

## Discussion

Les exostoses ostéocartilagineuses sont des excroissances osseuses recouvertes d'une coiffe cartilagineuse qui ne se développent que pendant la croissance. Malgré leur caractère congénital, les apparentant plus à des hamartomes, elles sont reprises parmi les tumeurs osseuses bénignes. Ces exostoses, appelées aussi ostéochondromes représentent 40% des tumeurs bénignes et 10% de l'ensemble des tumeurs osseuses primitives [1]. On connait deux formes cliniques: les exostoses solitaires et la maladie exostosante [2, 3]. La prévalence est de l'ordre de 0,9 à 2 par 100.000 habitants. Elles surviennent souvent chez les adolescents et rarement chez les nouveau-nés. Pour l'exostose solitaire, il n'y a pas de différence entre les deux sexes. Par contre, la maladie exostosante affecte plus fréquemment les hommes. Ces tumeurs se retrouvent préférentiellement sur le versant métaphysaire des cartilages de croissance fertiles. Elles restent souvent asymptomatiques. L'apparition de manifestations cliniques est en rapport avec la survenue des complications. Elles peuvent être extrinsèques par irritation ou compression d'une structure anatomique avoisinante; intrinsèques par la survenue d'une fracture de la base du pédicule ou une transformation maligne; ou mixtes secondaires à des déformations osseuses [3]. Les structures comprimées par l'ostéochondrome sont musculo-squelettiques [4], vasculaires ou neurologiques. La friction chronique du muscle ou du tendon peut produire une bursite, qui peut devenir infectée, hémorragique ou subir un changement métaplasique à l'origine des douleurs et du gonflement. Ce dernier tableau peut coïncider avec des transformations malignes en chondrosarcome [5]. D'autres complications des parties molles ont été rapportées tel que les ténosynovites, les ruptures tendineuses et les raideurs articulaires [6]. Les complications artério-veineuses secondaires aux exostoses peuvent toucher n'importe quel vaisseau. Mais la complication vasculaire la plus fréquente d'une exostose fémorale intéresse le segment distal de l'artère fémorale superficielle et l'artère poplité. Le pseudo-anévrysme reste la complication vasculaire la plus fréquente, souvent associé aux ostéochondromes sessiles, qui exercent plus des forces de friction sur le vaisseau, par comparaison aux ostéochondromes pédiculés [7]. Les complications nerveuses dépendent de la localisation des exostoses. Il s'agit d'un tableau de neuropathie, de radiculopathie ou de compression médullaire, dues aux exostoses localisées autour du genou et en regard de la moelle [8]. Les fractures intéressent les ostéochondromes pédiculés, rapportées au niveau du tibia proximal, moins fréquemment au niveau du stéo distal et rarement au niveau de l'humérus proximal. C'est une cause inhabituelle de stéoch de genou. Presque les stéo-quarts des cas guériront avec un traitement conservateur. Le risque de dégénérescence est de 1-2% dans l'exostose solitaire [9] et de 10 à 20% dans la maladie exostosante [10]. La transformation maligne survient dans la coiffe cartilagineuse et ne pose en général que peu de problèmes diagnostiques. Les chondrosarcomes périphériques prédominent chez l'adulte entre 30 et 60 ans. L'apparition de certains signes doivent faire redouter une transformation maligne, à savoir l'augmentation de la taille de la tumeur; l'apparition d'une ostéolyse; l'aspect flou des bords de l'exostose; la présence de calcifications en dehors de l'ossification principale; l’érosion de l'os porteur ou de l'os voisin; une épaisseur de plus de 1cm de la coiffe de cartilage et l'hyperfixation scintigraphique chez l'adulte. La présence d'un seul de ces signes doit conduire à une exérèse de type carcinologique.

## Conclusion

Les ostéochondromes sont des tumeurs osseuses bénignes fréquentes, souvent asymptomatiques. Elles peuvent devenir symptomatiques en cas de complications musculo-squelettiques, vasculaires, neurologiques ou de transformation métaplasique. Le risque de dégénérescence sarcomateuse nécessite une surveillance clinique et radiologique rapprochée et une exérèse chirurgicale au moindre doute.
